# A Genome-Wide Association Study of Outcome After Aneurysmal Subarachnoid Haemorrhage: Discovery Analysis

**DOI:** 10.1007/s12975-022-01095-4

**Published:** 2022-10-20

**Authors:** Ben Gaastra, Sheila Alexander, Mark K. Bakker, Hemant Bhagat, Philippe Bijlenga, Spiros L. Blackburn, Malie K. Collins, Sylvain Doré, Christoph J. Griessenauer, Philipp Hendrix, Eun Pyo Hong, Isabel C. Hostettler, Henry Houlden, Koji IIhara, Jin Pyeong Jeon, Bong Jun Kim, Jiang Li, Sandrine Morel, Paul Nyquist, Dianxu Ren, Ynte M. Ruigrok, David Werring, Will Tapper, Ian Galea, Diederik Bulters

**Affiliations:** 1 Faculty of Medicine, University of Southampton, Southampton, SO17 1BJ UK; 2 Department of Neurosurgery, Wessex Neurological Centre, University Hospital Southampton, Southampton, SO16 6YD UK; 3 School of Nursing, University of Pittsburgh, 3500 Victoria Street, Pittsburgh, PA 15261 USA; 4 Department of Neurology, University Medical Center Utrecht Brain Center, University Medical Center Utrecht, Heidelberlaan 100, 3584 CX Utrecht, the Netherlands; 5 Division of Neuroanaesthesia, Department of Anaesthesia and Intensive Care, Postgraduate Institute of Medical Education and Research (PGIMER), Chandigarh, India; 6 Neurosurgery Division, Department of Clinical Neurosciences, Faculty of Medicine, Geneva University Hospitals, Geneva, Switzerland; 7 University of Texas Houston Health Science Center, Houston, TX USA; 8 Geisinger Commonwealth School of Medicine, Scranton, PA USA; 9 Departments of Anesthesiology, Neurology, Psychiatry, Pharmaceutics, and Neuroscience College of Medicine, Center for Translational Research in Neurodegenerative Disease, McKnight Brain Institute, University of Florida, Gainesville, FL USA; 10 Department of Neurosurgery, Geisinger, Danville, PA USA; 11 Department of Neurosurgery, Paracelsus Medical University, Christian-Doppler Klinik, Salzburg, Austria; 12 Department of Neurosurgery, Saarland University Medical Center, Homburg, Germany; 13 Institute of New Frontier Research, Hallym University College of Medicine, Chuncheon, South Korea; 14 Stroke Research Centre, Institute of Neurology, University College London, London, UK; 15 Department of Neurosurgery, Kantonsspital St. Gallen, St. Gallen, Switzerland; 16 National Cerebral and Cardiovascular Center Hospital, 6-1 Kishibe-Shimmachi, Suita, Osaka Japan; 17 Department of Neurosurgery, Hallym University College of Medicine, Chuncheon, South Korea; 18 Department of Molecular and Functional Genomics, Weis Center for Research, Geisinger Health System, Danville, Danville, PA 17822 USA; 19 Department of Pathology and Immunology, Faculty of Medicine, University of Geneva, Geneva, Switzerland; 20 Departments of Neurology, Johns Hopkins School of Medicine, Baltimore, MD 21287 USA

**Keywords:** Subarachnoid haemorrhage, Stroke, Outcome assessment, Health care, Genetics, Medical

## Abstract

Candidate gene studies have identified genetic variants associated with clinical outcomes following aneurysmal subarachnoid haemorrhage (aSAH), but no genome-wide association studies have been performed to date. Here we report the results of the discovery phase of a two-stage genome-wide meta-analysis of outcome after aSAH. We identified 157 independent loci harbouring 756 genetic variants associated with outcome after aSAH (*p* < 1 × 10^−4^), which require validation. A single variant (rs12949158), in *SPNS2*, achieved genome-wide significance (*p* = 4.29 × 10^−8^) implicating sphingosine-1-phosphate signalling in outcome after aSAH. A large multicentre international effort to recruit samples for validation is required and ongoing. Validation of these findings will provide significant insight into the pathophysiology of outcomes after aSAH with potential implications for treatment.

**Supplementary Information:**

The online version contains supplementary material available at 10.1007/s12975-022-01095-4.

## Introduction

Aneurysmal subarachnoid haemorrhage (aSAH) is a devastating form of stroke with the worst outcomes and highest socioeconomic burden of any stroke type [1]. The pathophysiology of neurological injury following aSAH is incompletely understood. The mechanism is thought to be multifactorial with the initial surge in intracranial pressure following haemorrhage combined with the presence of blood breakdown products in the cerebrospinal fluid leading to a pattern of injury characterised by inflammation, cerebral vasospasm, microthrombosis, oxidative injury and cortical spreading depression [2–4]. Despite multiple clinical trials, nimodipine is the only therapeutic agent to improve outcomes [5]. It is our incomplete understanding of the mechanisms underlying neurological injury that is, at least in part, responsible for the lack of therapeutic innovation to improve outcomes.

The best outcome prediction model after aSAH, utilising clinical, demographic and imaging characteristics only explains up to 31% of the variation in outcome following aSAH [6]. Consequently, a large proportion of variation in outcome following aSAH is unexplained. There is a growing body of evidence from candidate gene studies that genetic background accounts for a proportion of this unexplained variation [7, 8]. However, no genome-wide analysis has been performed. Such a study would have the potential to provide valuable insights into the mechanisms underlying neurological injury following aSAH by identifying, as yet unstudied, genes associated with outcome and thus novel targets for therapeutic intervention.

In 2018, the HATCH consortium highlighted the need to better understand the pathophysiological mechanisms underlying outcome and proposed a large multicentre genetic analysis of outcome following aSAH [2]. The HATCH consortium has developed this proposal into an international collaboration to undertake a two-stage (discovery and validation) genome-wide association (GWA) study of outcome following aSAH, the protocol for which was published in this journal [9].

The aim of this manuscript is to (1) report the completion of the discovery stage of the study including preliminary results and (2) raise awareness of the study to recruit further samples for the validation stage.

## Methods

This is a two-stage (discovery and validation) GWA meta-analysis of outcome following aSAH. The results of the discovery analysis are reported in this manuscript. All analyses were performed according to the published protocol [9]. The study has both national ethical (REC 19 SC 0485) and institutional (ERGO 49253) approval.

For the discovery analysis, individuals were identified from (1) six studies from the HATCH consortium network and (2) the UK Biobank, a major biomedical database with extensive genetic and clinical data, previously described in detail[10] (application number 49305).

In the HATCH dataset, the primary outcome was the modified Rankin Scale (mRS) [11, 12] or Glasgow Outcome Scale (GOS) [13, 14] dichotomised into good (mRS 0–2, GOS 4–5) and poor (mRS 3–6, GOS 1–3) outcomes in the first two years following aSAH. The mRS and/or GOS are not available in the UK Biobank and, therefore, a measure of cognitive performance, psychomotor reaction time, was used since cognition is highly correlated with mRS/GOS following aSAH [15] and reaction time is significantly slower in aSAH cases compared to controls in the UK Biobank [16]. Reaction times were ranked from fastest to slowest and then the UK Biobank was dichotomised into good (faster) and poor (slower) outcomes, generating an equivalent proportion of good outcome individuals to the HATCH dataset.

Genotype information from eligible patients underwent quality control and imputation as required (see protocol for details [9]). Within individual cohorts, genetic variants were tested for association with dichotomised outcome using multivariable logistic regression under an additive model, controlling for confounding variables (age and genetic ancestry). A fixed effects meta-analysis was performed to determine each genetic variant’s overall effect size and significance. The meta-analysis was performed on all datasets and repeated in the HATCH dataset alone (i.e. excluding the UK Biobank given its alternative outcome metric). Independent loci were identified for validation using a clumping procedure to group single nucleotide polymorphisms (SNPs) in linkage disequilibrium (LD) (*R*^2^ > 0.2) and within 250 kb of each other. Index SNPs with suggestive significance (*p* < 1 × 10^−4^) were selected for validation. The threshold for genome-wide significance was *p* ≤ 5 × 10^−8^ and all analyses were performed using PLINK, STATA (StataCorp. 2011. Stata Statistical Software: Release 16. College Station, TX: StataCorp LP), wANNOVAR [17] and FUMA [18].

## Results

A total of 2489 samples were used for the discovery analysis following quality control. These samples were drawn from six datasets from the HATCH consortium [19–22] (*n* = 1685 patients) and 804 individuals from the UK Biobank. After dichotomisation of mRS/GOS within the HATCH consortium data, 1382 (82.0%) patients were classified as good outcome and 303 (18.0%) as poor outcome. Based on reaction times in the UK Biobank, 653 (81.2%) individuals were classified as good outcome and 151 (18.8%) as poor outcome. Table 1 details the demographics and other characteristics of the included datasets.

**Table 1:** Demographics and outcome data for included samples divided by dataset. If both mRS and GOS were available for a single study, the scale with greater data availability was used. If data availability was equal for mRS and GOS then mRS was used as it is the preferred outcome scale in stroke research. *WFNS*, World Federation of Neurological Surgeons; *SD*: standard deviation; ms: millisecond

	HATCH dataset						UK Biobank
Dataset	1	2	3	4	5	6	7
Origin	GOSH, UK	Utrecht, Netherlands	Geneva, Switzerland	Hallym, South Korea	Pittsburgh, USA	Geisinger, USA	UK Biobank
Selected publication detailing dataset	Bakker et al	Bakker et al	Bakker et al	Hong et al	Kim et al	Li et al	www.ukbiobank.ac.uk/enable-your-research/about-our-data/genetic-data
Sample size (*n*)	817	470	63	89	180	66	804
Age							
Mean (± SD)	54.4 (± 12.4)	57.2 (± 12.9)	53.6 (± 13.0)	58.7 (± 11.6)	54.9 (± 11.1)	57.7 (± 13.2)	47.6 (± 11.5)
Sex							
Male (*n*, %)	230 (28.2%)	129 (27.4%)	15 (23.8%)	32 (36.0%)	55 (30.6%)	21 (31.8%)	336 (41.8%)
Female (*n*, %)	587 (71.8%)	341 (72.6%)	48 (76.2%)	57 (64.0%)	125 (69.4%	45 (68.2%)	468 (58.2%)
WFNS grade (*n*, %)					*Hunt and Hess*		
1	473 (57.9%)	216 (46.0%)	39 (61.9%)	48 (53.9%)	27 (15.0%)	30 (45.5%)	-
2	171 (20.9%)	127 (27.0%)	7 (11.1%)	1 (1.1%)	56 (31.1%)	12 (18.2%)	-
3	36 (4.4%)	23 (4.9%)	5 (7.9%)	10 (11.2%)	61 (33.9%)	4 (6.1%)	-
4	88 (10.8%)	57 (12.1%)	5 (7.9%)	20 (22.5%)	23 (12.8%)	8 (12.1%)	-
5	42 (5.1%)	44 (9.4%)	7 (11.1%)	10 (11.2%)	13 (7.2%)	12 (18.2%)	-
Missing	7 (0.9%)	3 (0.6%)	-	-	-	-	804 (100.0%)
Time to follow-up (months)							
Mean (± SD)	9.4 (± 6.8)	3.0 (± 0)	6.5 (± 2.5)	5.6 (± 1.2)	6.0 (± 2.2)	3.0 (± 0)	129.3 (± 122.1)
Missing (*n*, %)	89 (10.9%)	-	-	-	-		-
Outcome							
mRS							
0 (*n*, %)	260 (31.8%)	5 (1.1%)	6 (9.5%)	0 (0.0%)	43 (23.9%)	5 (7.6%)	-
1 (*n*, %)	300 (36.9%)	26 (5.5%)	19 (30.2%)	48 (53.9%)	64 (35.6%)	22 (33.3%)	-
2 (*n*, %)	134 (16.5%)	319 (67.9%)	18 (28.6%)	9 (10.1%)	27 (15.0%)	18 (27.3%)	-
3 (*n*, %)	59 (7.2%)	20 (4.3%)	12 (19.0%)	15 (16.9%)	9 (5.0%)	6 (9.1%)	-
4 (*n*, %)	24 (2.9%)	33 (7.0%)	1 (1.6%)	7 (7.9%)	3 (1.7%)	4 (6.1%)	-
5 (*n*, %)	11 (1.4%)	30 (6.4%)	0 (0.0%)	3 (3.4%)	5 (2.8%)	12 (18.2%)	-
6 (*n,* %)	0 (0.0%)	37 (7.9%)	7 (11.1%)	7 (7.9%)	29 (16.1%)	0	-
Missing (*n*, %)	29 (3.6%)	-	-	-	-	-	-
GOS							
1 (*n*, %)	31 (3.8%)	37 (7.9%)	-	7 (7.9%)	29 (16.1%)	-	-
2 (*n*, %)	0 (0.0%)	1 (0.2%)	-	2 (2.2%)	0 (0.0%)	-	-
3 (*n*, %)	33 (4.1%)	62 (13.2%)	-	11 (12.4%)	10 (5.6%)	-	-
4 (*n*, %)	124 (15.2%)	123 (26.2%)	-	13 (14.6%)	42 (23.3%)	-	-
5 (*n*, %)	629 (77.3%)	247 (52.6%)	-	56 (62.9%)	99 (55.0%)	-	-
Missing (*n*, %)	-	-	-	-	-	-	-
Reaction time (ms)							
Mean (± SD)	-	-	-	-	-	-	587.2 (± 138.3)
Missing	-	-	-	-	-	-	-
* Metric definingout come*	*GOS*	*mRS*	*mRS*	*mRS*	*mRS*	*mRS*	*Reaction time*
Good (*n*, %)	753 (92.5%)	350 (74.0%)	43 (68.3%)	57 (64.0%)	134 (74.4%)	45 (68.1%)	653 (81.2%)
Poor (*n*, %)	64 (7.9%)	120 (26.0%)	20 (31.7%)	32 (36.0%)	46 (25.6%)	21 (31.8%)	151 (18.8%)

**Fig. 1:**
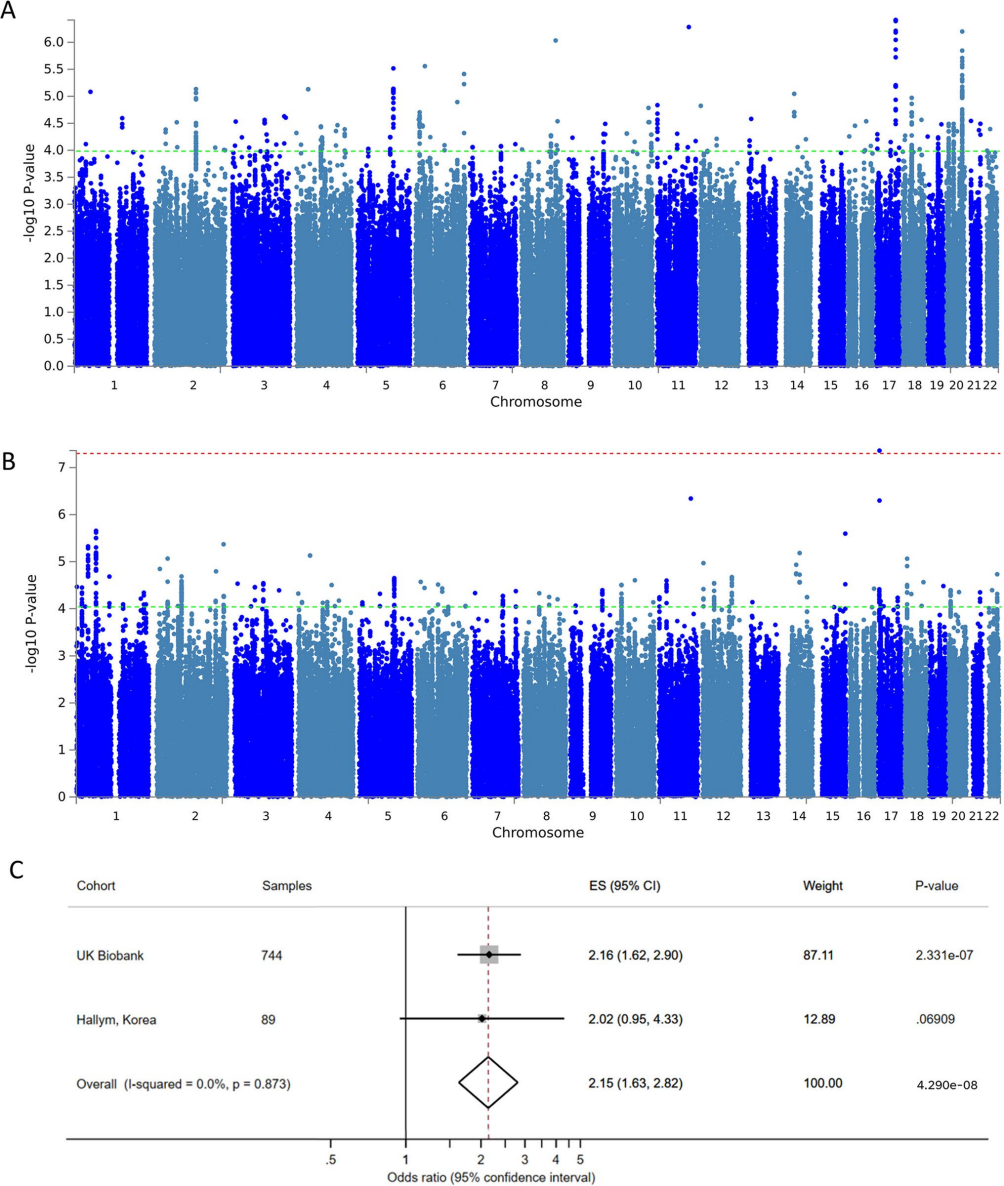
**A** Manhattan plot from the meta-analysis that includes HATCH datasets alone. **B** Manhattan plot from the meta-analysis that includes UK Biobank and HATCH datasets. The red dotted line signifies genome-wide significance (*p* < 5 × 10^−8^), green dotted line signifies suggestive significance (*p* < 1 × 10.^−4^). Manhattan plots generated using FUMA. **C** Forest plot for genome-wide significant SNP rs12949158. rs12949158 genotypes in: UK Biobank: AA 252, AG 346, GG 146; Korean dataset: AA 22, AG 42, GG 25

Analysis of samples from the HATCH consortium (*n* = 1685) identified 403 SNPs associated with clinical outcome (*p* < 1 × 10^−4^) within 97 independent loci after LD-based SNP clumping (Fig. 1A and Supplementary Table 1A and B). No genetic variants reached genome-wide significance.

Including all seven datasets (*n* = 2489) 85 independent loci were identified from 406 SNPs, associated with clinical outcome (*p* < 1 × 10^−4^) (Fig. 1B and Supplementary Table 2A and B). A single variant, rs12949158, reached genome-wide significance (*p* = 4.29 × 10^−8^). rs12949158 is located on chromosome 17 in an intronic region of the sphingolipid transporter 2 (*SPNS2*) gene, which codes for the major transporter of sphingosine-1-phosphate (S1P) (Supplementary Table 2A). The rs12949158 SNP was only genotyped in two datasets [UK Biobank (*n* = 744) and Korean datasets (*n* = 89)] (Fig. 1C). Arrays used in the other datasets did not include rs12949158 or any other SNPs with sufficient LD to allow reliable imputation. The rs12949158 alternate A allele was associated with an increased risk of poor outcome with an odds ratio of 2.15 (95% confidence interval 1.63–2.82). The association of rs12949158 with psychomotor reaction time was specific to aSAH, since in a previously published UK Biobank control cohort matched to the same aSAH population[16], this relationship was absent (*p* = 0.55).

Including both analyses, a total of 157 independent loci were identified from 756 unique SNPs and these will be taken forward for validation.

## Discussion

In this discovery genome-wide meta-analysis, we identified 157 independent loci from 756 unique SNPs associated with outcome following aSAH (*p* < 1 × 10^−4^) for validation. We also report that the rs12949158 alternate A allele, located within the *SPNS2* gene, was associated with an increased risk of poor outcome after aSAH (OR 2.15 95% CI 1.63–2.82) with genome-wide significance (*p* = 4.29 × 10^−8^). Although one possible alternative explanation is that rs12949158 associates with psychomotor reaction time independent of aSAH, this association was not observed in control individuals in the UK Biobank.

The genome-wide significant rs12949158 finding is not conclusive and requires validation. Firstly, the rs12949158 genotype was only typed in a subset of the discovery cohort (*n* = 833). Secondly, the finding is primarily driven by the UK Biobank, which uses an outcome measure that is different from the other datasets (psychomotor reaction time). In the second stage, a customised genotyping array will be used to directly capture all variants targeted for validation including rs12949158.

The rs12949158 variant is intronic, located within the gene *SPNS2*, a member of the S1P signalling pathway. While variation in a gene intron does not guarantee that the same gene is involved, intron-mediated enhancement of gene expression is increasingly recognised and the intronic variation is most likely to regulate the closest gene [23]. Moreover, the S1P signalling pathway is a biologically plausible candidate to influence outcomes after aSAH since S1P has been implicated in neurological injury following stroke via activation of S1P receptors (S1PR) leading to microglial activation, neuronal death, inflammation and blood–brain barrier disruption [24, 25]. Specifically, after human aSAH, S1P was found to be elevated in the cerebrospinal fluid where its concentration correlated with haemorrhage volume [26] and worse neurological outcome [26]. A possible mechanism linking S1P to clinical outcome is provided by studies showing that S1P induces cerebral vasospasm in canine basilar artery in vitro and in vivo [27] and in murine basilar artery in vitro via S1PR3 [28]. This pathway is of particular interest since S1PR-modulating drugs, already licensed in other neurological conditions (e.g. fingolimod) have been shown to be neuroprotective in ischemic stroke [29] and intracerebral haemorrhage [30] in humans and could be re-purposed for aSAH if this finding is validated.

The study’s population was biased towards participants with a good outcome almost certainly because individuals with a poor prognosis were less likely to be recruited. However, this study’s aim was to better understand the pathophysiological mechanisms underlying outcomes in survivors with a view to developing treatments to improve outcomes. Individuals dying in the acute phase or early after admission will unfortunately be unlikely to benefit from such interventions. Hence, while this study was biased towards participants with a good outcome, these are the individuals most likely to benefit from the findings.

This discovery study achieved its prespecified sample size in a timely manner and represents a highly successful international collaboration generating interesting results to take forward to validation. The total target sample size including the validation cohort is 5000 [9] which would be powered to detect common variation (minor allele frequency (MAF = 0.4) with an effect size of 1.39 and rare variation (MAF = 0.1) with an effect size of 1.66 at genome-wide significance. Recruitment is ongoing for validation and investigators wishing to collaborate using either retrospective or prospective data can find further information on the study website. The study has been designed to maximise inclusivity. It is open to any investigator able to provide the following de-identified biosamples or data from patients with aSAH: genome-wide genotype information (or DNA/cellular sample for genotyping), mRS/GOS within two years of haemorrhage, age, sex and evidence of institutional review board approval. In addition, funding will be provided to facilitate genotyping where local funding is not already in place. Where data availability allows we will also explore whether significant genetic variants mediate the effect of other factors, known to influence outcome after aSAH such as clinical and radiological features.

## Supplementary Information

Below is the link to the electronic supplementary material.

Supplementary file1 (XLSX 159 KB)

## Data Availability

Study data will be available from the authors subject to institutional agreements and ethical approvals.
